# 881 Evaluation of High-Dose, Once-Daily Tobramycin Dosing in Patients with Thermal Injury

**DOI:** 10.1093/jbcr/iraf019.412

**Published:** 2025-04-01

**Authors:** Asia Quan, Monika Zmarlicka, Curt Bay, James Sanders, Karen Richey, Kevin Foster

**Affiliations:** Diane & Bruce Halle Arizona Burn Center; Diane & Bruce Halle Arizona Burn Center; A.T. Still University; University of Texas Southwestern; Diane & Bruce Halle Arizona Burn Center; Diane & Bruce Halle Arizona Burn Center

## Abstract

**Introduction:**

Patients with thermal injury undergo metabolic changes resulting in a hypermetabolic state, leading to altered pharmacokinetics in antimicrobials, including tobramycin. Tobramycin efficacy is associated with peak to minimum inhibitory concentration (MIC) ratio of 8-10; thus, for an MIC of 2, ideal minimum tobramycin peaks are between 16-20 mcg/mL. Optimal tobramycin dosing strategies in those with thermal injuries are undefined, as literature suggests large variations in kinetics that may require higher doses compared to standard dosing. Due to previous evaluations at our institution showing that 7 mg/kg tobramycin dosing resulted in insufficient kinetic parameters, standard dosing for those with thermal injury was increased to 10 mg/kg. The main objective of this study is to describe kinetics associated with this new dosing strategy.

**Methods:**

Retrospective chart review of patients receiving tobramycin from January 2016 to August 2017. Patients at least 18 years old with thermal injury who received tobramycin 10 mg/kg q24h and had two tobramycin levels available evaluation of tobramycin kinetics were included in the study.

**Results:**

A total of 68 tobramycin regimens from 47 patients were included in the study. Patients had a mean total burn surface area of 34.4% ± 21.8%. Mean calculated tobramycin kinetic parameters were as follows: peak 16.9 mcg/mL ± 8.2; volume of distribution 0.62 L/kg ± 0.31; half-life 4.9 hr ± 4.6; ke 0.19 ± 0.07; time to level < 2 mcg/mL 15.7 hr ± 16.7. In 28 (41.1%) regimens, the tobramycin peak was at least 16 mcg/mL. On generalized estimating equations binomial analysis, age (OR=1.069 [96% CI 1.012 – 1.128]) and number of days post burn injury tobramycin initiated (OR=1.023 [95% CI 1.001 – 1.045]) were found to be significantly associated with increased likelihood of achieving a tobramycin peak of at least 16 mcg/mL.

**Conclusions:**

A 10 mg/kg q24h tobramycin dosing is a reasonable dosing strategy in patients with thermal injury, as mean peaks associated with this dosing strategy would be considered therapeutic. Kinetics remained variable, and age and time from initial thermal injury were found to be related to likelihood of achieving a therapeutic peak; thus therapeutic monitoring and dose adjustments based on individualized kinetics is recommended.

**Applicability of Research to Practice:**

This study provides additional insights not previously reported in the literature to help guide tobramycin dosing in those with thermal injury.

**Funding for the Study:**

N/A

